# Dangerous Pressurization and Inappropriate Alarms during Water Occlusion of the Expiratory Circuit of Commonly Used Infant Ventilators

**DOI:** 10.1371/journal.pone.0154034

**Published:** 2016-04-26

**Authors:** Murray Hinder, Aldo Perdomo, Mark Tracy

**Affiliations:** 1 Neonatal Intensive Care Unit, Westmead Hospital, Sydney, New South Wales, Australia; 2 Sydney University, Sydney, New South Wales, Australia; 3 Department of Paediatrics and Child Health Sydney University, Sydney, New South Wales, Australia; Azienda Ospedaliero-Universitaria Careggi, ITALY

## Abstract

**Background:**

Non-invasive continuous positive airways pressure is commonly a primary respiratory therapy delivered via multi-purpose ventilators in premature newborns. Expiratory limb occlusion due to water accumulation or ‘rainout’ from gas humidification is a frequent issue. A case of expiratory limb occlusion due to rainout causing unexpected and excessive repetitive airway pressurisation in a Draeger VN500 prompted a systematic bench test examination of currently available ventilators.

**Objective:**

To assess neonatal ventilator response to partial or complete expiratory limb occlusion when set to non-invasive continuous positive airway pressure mode.

**Design:**

Seven commercially available neonatal ventilators connected to a test lung using a standard infant humidifier circuit with partial and/or complete expiratory limb occlusion were examined in a bench test study. Each ventilator was set to deliver 6 cmH_2_O in non-invasive mode and respiratory mechanics data for 75%, 80% and 100% occlusion were collected.

**Results:**

Several ventilators responded inappropriately with complete occlusion by cyclical pressurisation/depressurisation to peak pressures of between 19·4 and 64·6 cm H_2_O at rates varying between 2 to 77 inflations per minute. Tidal volumes varied between 10·1 and 24·3mL. Alarm responses varied from ‘specific’ (tube occluded) to ‘ambiguous’ (Safety valve open). Carefusion Avea responded by continuing to provide the set distending pressure and displaying an appropriate alarm message. Draeger Babylog 8000 did not alarm with partial occlusions and incorrectly displayed airways pressure at 6·1cmH_2_O compared to the measured values of 13cmH_2_O.

**Conclusions:**

This study found a potential for significant adverse ventilator response due to complete or near complete expiratory limb occlusion in CPAP mode.

## Introduction

A recent adverse ventilator event occurred in our unit with a stable extremely preterm infant managed on 6 cmH_2_O nasal continuous positive pressure (nCPAP) delivered via a Draeger VN500 (Lübeck, Germany). This male infant was born at 28 weeks gestation with a birth weight of 1000grams. After a brief period of intubation for Respiratory Distress Syndrome and treatment with surfactant (Curosurf, Chiesi Farmaceutici, S.p.A., Parma—Italy), he was extubated successfully to nCPAP. Sudden deterioration in the patient condition was observed with desaturation and bradycardia. The ventilator pressure waveform indicted cyclical pressurisation to a peak of 40cmH_2_O falling to zero at a rate of approximately 77 inflations per minute and alarm message indicating high/low pressures. Patient was immediately taken off the ventilator circuit and provided with manual mask ventilation using a t-piece resuscitator. The infant recovered quickly and the ventilator circuit was subsequently discovered to have complete expiratory limb occlusion with water due to rainout in the opaque circuit. Given the high pressures observed, we regarded this “near miss” event as potentially life threatening. The VN500 in question and another 4 similar VN500 ventilators were tested to determine no faults in standard function yet all responded to complete expiratory limb occlusion in the same manner of cyclical pressurisation/depressurisation.

In searching the literature we were surprised to find a detailed case report published over thirty years ago by Hall[1] et al (1983) describing a similar complication of prolonged excessive airway pressure during circuit occlusion where the infant died. Following this event, Hall[1] et al bench tested 8 continuous flow, pressure-limited infant ventilators available at that time, examining response to complete expiratory limb occlusion. This study also assessed the specifications and design of the inspiratory pressure regulating valve of these ventilators. Hall[1] found in this study, the majority of infant ventilators tested exposed the patient to excessive and inadvertent airway pressures well over the set values on the ventilator during the occlusion.

Given the potential seriousness of this problem we planned to systematically test a range of neonatal ventilators commercially available and currently in use internationally. The aim of this study was to examine ventilator alarm response and pressure/flow changes when states of partial or complete expiratory limb occlusion were induced in a bench test setting for a range of neonatal ventilators set in non-invasive CPAP mode.

## Background

The manufacture and software control of neonatal ventilators has increased markedly in the last decade. Many of the latest generation of neonatal ventilators are multifunction devices that offer both invasive (patient circuit to endotracheal tube) and non-invasive mechanical ventilation (patient circuit to nasal prongs or mask). The gas flow delivered to the patient circuit has changed from constant flow to a proportional or demand flow in some brands and modes. This is common in adult ventilators but relatively new in the neonatal designs. Managing complex patient—ventilator interactions such as volume targeting and breath termination as well as monitoring alterations in delivered ventilation is becoming more complex[2] and demands a considerable level of clinical expertise.[3,4]

Mechanical ventilation in the neonatal intensive care unit environment has additional complexity due to widespread use of closed incubators used for thermoregulation. Exposure to expiratory limb tube kinking can occur with soft tube CPAP delivery hardware (midline delivery manifolds) and incubator doors. Water build up (rainout) in ventilator circuits due to the humidification process is common and the smaller diameter of neonatal circuits thus poses greater risk of partial or total occlusion. The amount of rainout and the time it takes to accumulate will vary depending on many factors (ventilator circuit type and design, incubator temperature,[5] nursery temperature,[6] circuit orientation (particularly the diameter of any downward facing loops in the expiratory limb tubing) and ventilator delivered tidal volume[7]). Ventilators are designed to cope with a wide range of potential error states including ‘rainout’ in ventilator circuits. The potential for partial or even total expiratory limb occlusion is significant, therefore heated patient circuits and evaporative expiratory systems are designed to minimize this problem. Due to the range of patient size, gestation and postnatal age, the location of the patient circuit temperature probe either in or outside a closed incubator is critical to prevent excessive circuit rainout. [5,8,9]

The paper by Hall[1] et al published over thirty years ago reported a serious systematic design fault in neonatal ventilator systems available at that time. We aimed to bench test the currently available neonatal ventilators using similar methods to those used by Hall[1] et al to determine if the previously identified design faults still exist in current ventilators. We also wished to determine if current international standards[10] were appropriate for ventilator delivered non-invasive CPAP.

## Materials and Method

### Sample

Seven commercially available neonatal ventilators were tested.

VN500 (Draeger, Lubeck Germany)

Fabian (Acoutronic, Hirzel Switzerland),

SLE 5000 (SLE Ltd, South Croydon United Kingdom),

Babylog 8000 (Draeger, Lubeck Germany).

Avea (Carefusion, San Diego USA),

Leoni (Heinen + Lowenstein, Bad Kissingen Germany)

Sophie (Stephan Medizintechnik, Gakenbach Germany).

A Fisher & Paykel RT265 infant humidifier circuit was used on each ventilator except the Sophie ventilator which has a dedicated airway circuit and humidification system of different circuit diameter. (Thus the Sophie ventilator was only tested at 100% occlusion with operator adjusted over pressure valve manually set to its maximum value of 70cmH_2_O to simulate worst case).

### Bench Test

Partial flow restrictors were manufactured using two precision drill bits with diameters of 2·783mm (#33 US number drill bit) and 2·260mm (#43 US number drill bit) and mounted inside airway adaptors. These restrictors represent occlusion values of 75·54% and 80·14% of the measured internal expiratory limb circuit diameter. Internal diameter of expiratory limb tubing was measured at 11·38mm (+/-0·01mm). For 100% occlusion an airway adaptor containing a short section (length 80mm internal diameter 11·5mm) of exposed silicone tubing was used. Tubing was clamped with large artery forceps to achieve 100% occlusion, this was also tested against water insertion (approx. 20ml H_2_O) into expiratory circuit with tight (10cm across) downward facing circuit loop, with the same results. The humidifier base was not powered on. Humidifier canister was installed on the base and filled with water to the prescribed level. The patient circuit was connected to a test lung with known compliance of 0·5 mL/cm H_2_O (Draeger, Lubeck, Germany). The entire system was pressure checked and found to be leak free. All manufacturer recommended pre use checks and calibrations were carried out prior to selecting CPAP treatment mode set to deliver 6 cmH_2_O of patient airway pressure for each ventilator tested. If circuit flow needed to be set manually in CPAP mode it was set to 8 litres per minute (LPM).

Airway pressures and flows delivered to a neonatal test lung were measured at the patient 'y' connector by the pneumotach and pressure transducer (Florian respiratory monitor). Data for each occlusion value was collected separately for 2 minutes using data acquisition software (Spectra, Grove Medical) at a sampling frequency of 200Hz.

Ventilator alarm response and ventilator displayed mean airway pressure for each occlusion value was also recorded. Respiratory monitor volume calibration was performed with a syringe of known volume and pressure with a traceable reference electronic manometer (IMT Medical Model PF3000).

Draeger Australia was contacted following our initial Australian Therapeutic Goods (TGA) alert (Report No: 27809) with the observed VN500 occlusion behaviour with software version SO2·30. In response Draeger provided software (sw) revisions SO2·31 and SO2·41 with altered alarm message for the situation described. This software change also allows the user to adjust the maximum flow limit in non-invasive CPAP mode from a minimum of 6LPM to 30LPM (the default at system/ventilator initiation). The bench testing for VN500 was performed on original sw version SO2·30 with a maximum flow rate of 36LPM and sw version SO2·41 at user adjusted maximum flow rates of 10, 20 and 30 LPM.

This study was approved by the Western Sydney Local Health District Human Ethics and Scientific committee, approval number SAC2013/8/6·2 (3793).

## Results

Table 1 details the measured test lung respiratory data and ventilator response (MAP and alarm messages) by brand at each level of graded expiratory limb occlusion. Table 2 details changes between VN500 software versions SO2·30 and SO2·41.

**Table 1:** Measured test lung respiratory data and ventilator response by brand to occlusion, CPAP set to 6cmH_2_O.

		Test Lung Respiratory Values							Ventilator Response	
VentilatorBrand/Model	Occlusion %	Resp Rate*/min*	VTi *mL*	Insp *sec*	Exp *sec*	Peak *cmH_2_O*	PEEP/CPAP *cmH*_*2*_*O*	MAP *cmH*_*2*_*O*	MAP Displayed *cmH*_*2*_*O*	Alarm Message
**Draeger 'Babylog 8000'**sw ver: 5·01	75						10·0		6·1	None
	80						13·0		6·1	None
	100	7·4	10·5	2·5	5·5	19·4	2·4	7·4	0·0	Hose Kinked
**Draeger 'VN500'** sw ver: SO2·30 *(max flow limit 36 LPM)*	75						8·6		8·6	None
	80						10·9		11·0	None
	100	77·0	21·9	0·3	0·5	47·9	0·2	10·1	8·5	Airway Press high
**Carefusion 'Avea’** sw ver: 4·4	75						6·1		6·0	None
	80						6·1		6·0	None
	100						6·3		6·0	Circuit Occlusion
**Acutronic 'Fabian'** sw ver: 2·0·0·34	75						7·0		6·7	None
	80						12.6		12·3	High Pressure
	100	30·3	17·2	0·3	1·7	23·0	-0·1	2·6	_·_	High Pressure/Tube Occluded
**SLE '5000'** sw ver: 4·3	75						9·9		9·0	None
	80	6·5	12·0	4·3	5·2	17·9	0·7	8·4	8·0	Continuing Positive Pressure
	100	2	20·0	0·8	3·0	48·5	5·9	22·5	20·0	Continuing Positive Pressure/High Pressure
**Heinen + Lowenstein'Leoni Plus’** sw ver: 2·3·20	75	23·9	9·7	0·1	2·4	20·7	-0·3	0·6	0·0	High PEEP, Low PEEP
	80	24·6	9·8	0·2	2·3	20·7	-0·3	0·6	0·0	High PEEP, Low PEEP
	100	24·9	10·1	0·1	2·3	21·6	-0·3	0·7	0·0	High PEEP, Low PEEP
**Stephan 'Sophie'** sw ver: 12·2	100	5·0	24·3	8·1	4·0	64·6	6·1	30·3	9·0	Safety Valve Open

**Table 2:** Draeger VN500 software version comparison. CPAP set at 6cmH_2_O.

			Test Lung Respiratory Values							Ventilator Response	
Software *Ver No*	Flow limit *LPM*	Occlusion *%*	Resp Rate*/min*	VTi *mL*	Insp *sec*	Exp *sec*	Peak *cmH*_*2*_*O*	PEEP/CPAP *cmH*_*2*_*O*	MAP *cmH*_*2*_*O*	MAP Displayed *cmH*_*2*_*O*	Alarm Message(response time)
SO2·30	36	75						8·6		8·6	None
SO2·41	10	75						6·8		6·5	Airway Press low (50sec)
“	20	75						6·6		6·5	Airway Press low (50sec)
“	30	75						6·4		6·4	Airway Press low (12sec)
SO2·30	36	80						10·9		11	None
SO2·41	10	80						7·4		7·4	Airway Press low (5sec)
“	20	80						7·2		7·0	Airway Press low (5sec)
“	30	80						7·2		7·1	Airway Press low (10sec)
SO2·30	36	100	77·0	21·9	0·3	0·5	47·9	0·2	10·1	8·5	Airway Press high (0sec)
SO2·41	10	100	11·1	20·8	5·2	0·8	35·5	0·3	13·5	_·_	Airway Press high/low (0sec)
“	20	100	13·3	20·0	4·8	0·7	34·1	0·2	15·6	_·_	Airway Press high/low (0sec)
“	30	100	11·3	21·0	5·1	0·8	35·5	0·2	16·7	_·_	Airway Press high/low (0sec)

### Alarm Response

There was a wide variation in alarm response from no alarm state or message at expiratory limb occlusions of 75% to 80% occluded to ventilator systems showing an alarm message indicating a tube occlusion or obstruction. A “hose kinked” message was displayed with a 100% occlusion on the Babylog 8000, a “circuit occlusion” message occurred with a 100% occlusion with the Carefusion Avea and a “High pressure/Tube occlusion” message occurred with the Acutronics Fabian at 100% tube occlusion. The Draeger VN500 sw SO2·41 gave alarm message of airway pressure “low” at 75% and 80% occlusion even though the mean airway pressure was not low (6·4 to 6·8 cmH_2_O at 75% occlusion and 7·2 to 7·4 cm H_2_O at 80% occlusion) (Table 1).

### Pressure Response

**Fig 1:**
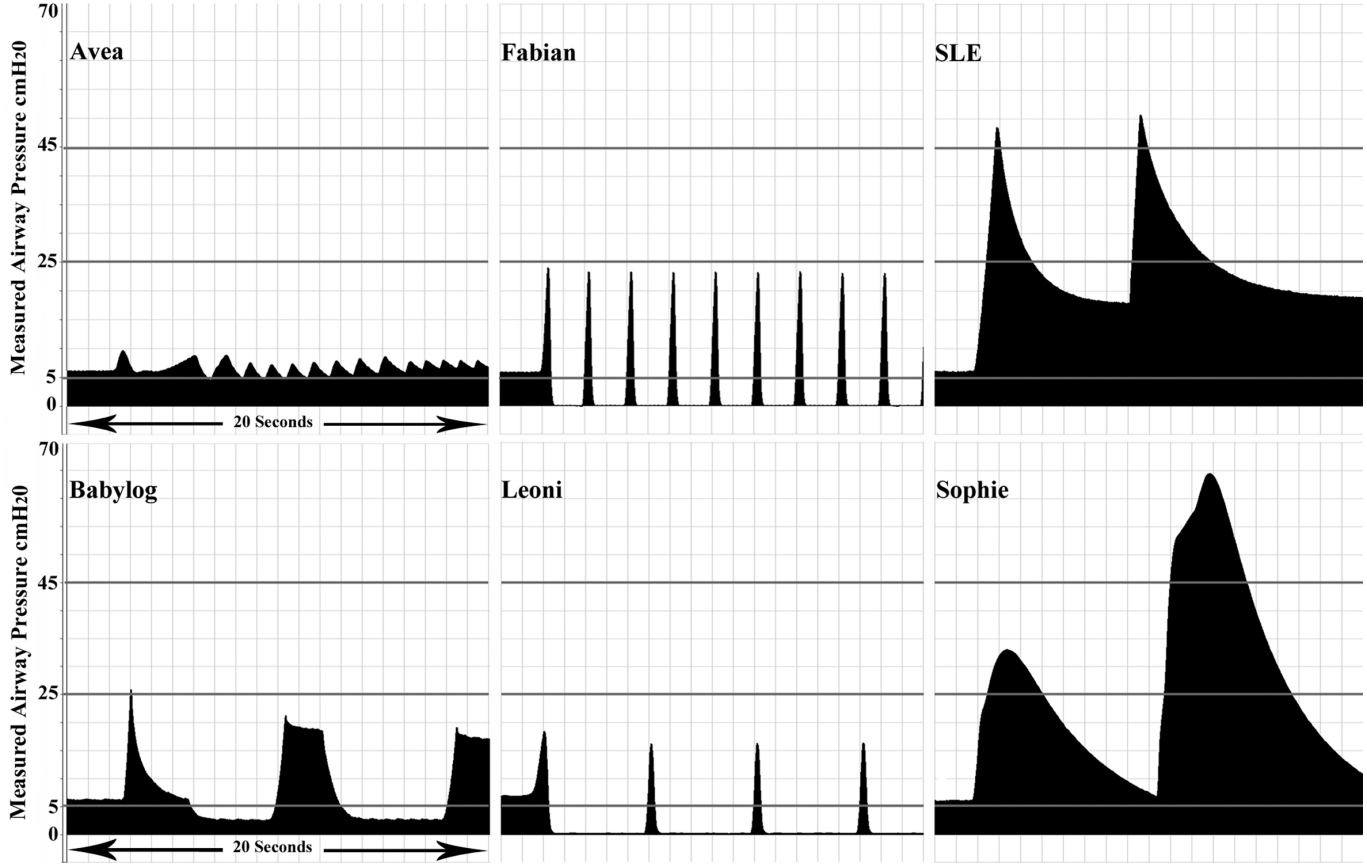
Measured airway pressure by brand to 100% occlusion, CPAP set to 6cmH_2_O.

**Fig 2:**
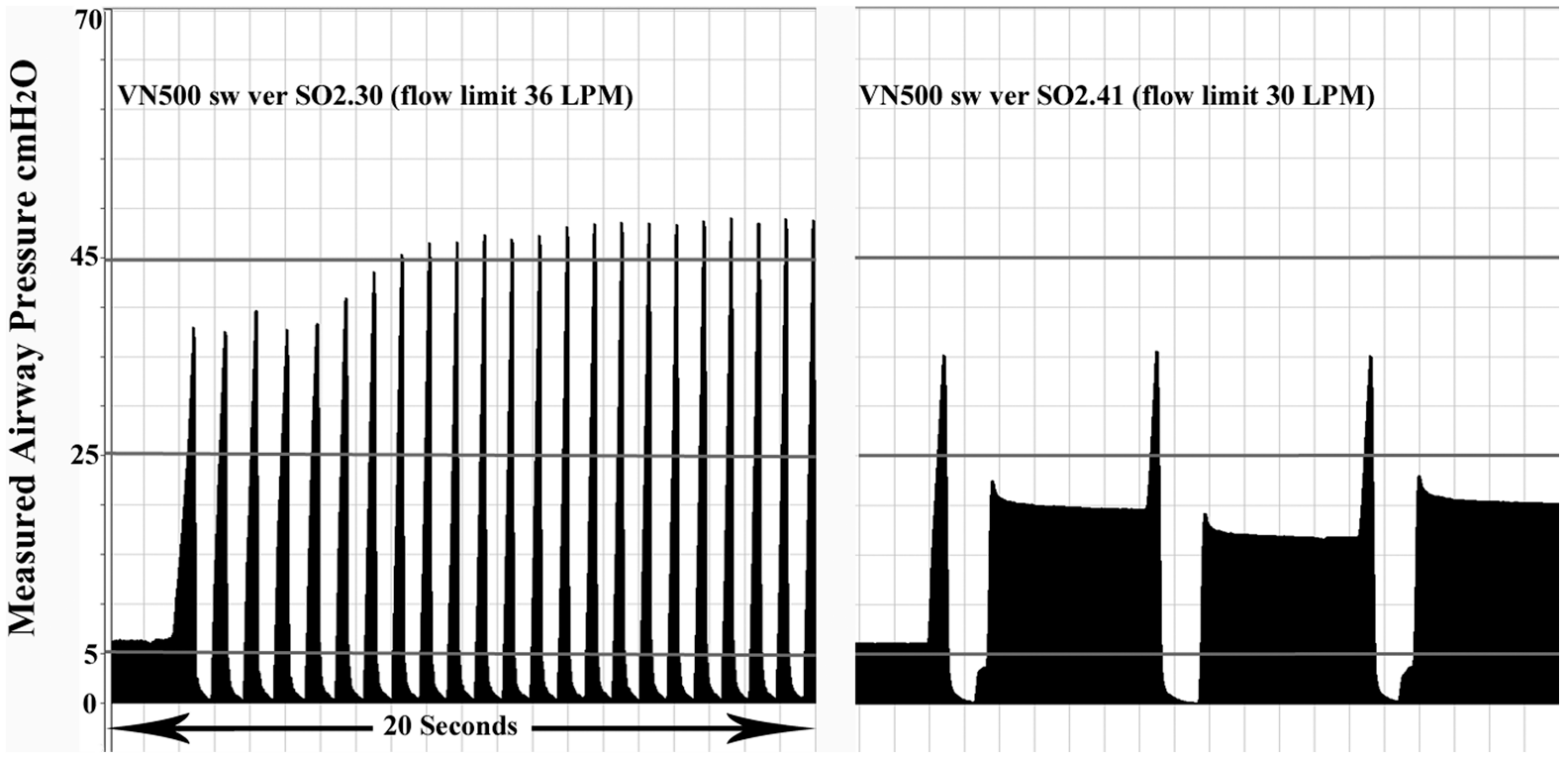
Drager VN500 measured airway pressure by software version to 100% occlusion, CPAP set at 6cmH_2_O.

There was also a wide variation in ventilator responses to levels of occlusion from either continuing to provide continuous distending airway pressure at the set pressure with 75%, 80% and 100% expiratory limb occlusion; to states where the ventilator cyclically pressurized then dumped in a manner similar to time cycled pressure ventilation (Fig 1) delivering large tidal volumes (range 9·7mL to 24·3mL) (Table 1). The rate of these cycles varied from slow (2–7 pressurizations per minute) to rates of 11 to 77 in the case of the two versions of the VN500 software (Table 2 and Fig 2). One system (the Heinen + Lowerstein Leoni plus ventilator) cycled from pressurization and dumping at all levels of partial and complete expiratory limb occlusion. The Draeger Babylog 8000 with software version 5·01 was noteworthy in not displaying the correct patient MAP with a measured patient value of 13cmH_2_O compared to the ventilator displayed, 6·1 cmH_2_O at 80% expiratory limb occlusion with no alarm. The peak airway pressure varied from 17·9cmH_2_O to 64·6 cmH_2_O regardless of the CPAP being set to 6cmH_2_O. Auto PEEP was not noted on any brand below 70% occlusion.

## Discussion

This study found that amongst the range of ventilators tested the delivery of non-invasive CPAP therapy during partial or total expiratory limb occlusion had the potential to expose the neonatal lung to pressures and volumes that are clinically unacceptable and dangerous.

The study is potentially limited by the fact that our bench test setup is not designed to simulate the clinical scenario where the infant may have been protected by mask leak where high pressures were delivered. Our bench test is designed as a ‘no leak’ system to examine the worst case scenario should the infant have no or minimal mouth leak as might occur with a chin strap and good nasal nares seal to prongs.[11,12] Our results may not be generalizable to other brands of mechanical ventilator or software revisions not tested.

Ventilators are designed with an inspiratory pressure regulator (IPR) in the expiratory limb exhalation block. This allows the regulation of pressures delivered to the patient at safe and repeatable levels selected by the clinician. Thirty two years ago Hall et al showed that partial or total occlusion between the patient 'y' and the exhalation block can alter or mitigate the IPR ability to function correctly.[1]

Carefusion Avea ventilator was the only ventilator tested, that behaved in a manner most clinicians’ would regard as appropriate. That is, to alarm with a message that would immediately suggest the appropriate corrective action and to continue to provide the set CPAP level, even with 100% occlusion.

Although rainout is minimised in the Fisher & Paykel RT265 circuit due to its unique design with an evaporative material (Evaqua^™^), it is not completely eliminated in the expiratory limb. Visualization of rainout content in the expiratory limb is difficult due to its opaque design. Product literature recommends six hourly inspection and removal of rainout if needed.[13]

There are two common situations where we have clinically seen patient events with excessive expiratory limb rainout causing complete occlusion with the Fisher & Paykel RT265 circuit.

The first was with accidental disconnection of expiratory limb heater during patient equipment movement. There is no alarm to indicate this with the Fisher & Paykel MR850 and can be undetected. In other types of humidifier circuits (particularly non Evaqua^™^ bubble nasal CPAP) without heated expiratory limbs, accumulation of expiratory limb rainout and delivered pressure may be increased.[14] The second situation is placing the Fisher & Paykel RT265 circuit temperature probe inside a closed incubator with high set temperature.[5,8,9] In this situation with an incubator temperature of greater than 34·0C the Fisher & Paykel humidifier drives excessive vaporised water producing rainout. The manufacturer recommends the use of an extension tube to site the circuit temperature probe outside the incubator.

The software alteration by Draeger (reflected in software revisions SO2·31 and SO2·41) in response allows the user to adjust the pre-set (and maximum) inflow rate of 30 LPM to a minimum of 6 LPM in the non-invasive CPAP delivery mode. The impact in our bench test only showed some improvement at 100% expiratory limb occlusion with the cyclical pressurization /airway pressure dumping rate dropped from 76 to 13 per minute (but with a distending inspiratory time increase from 0·3 to 5·2 seconds), the pressurization tidal volume decreased slightly from 21·9mL to 21mL and the peak pressurization pressure decreased from 47·9 cmH_2_O to 35·5 cmH_2_O. At 75% and 80% expiratory limb occlusions, the VN500 with sw SO2·41 gave ambiguous airway pressure message “airway pressure low” message after 50 seconds (75% occlusion at 10 and 20 LPM) and 10 seconds (80% occlusion at 30 LPM) with displayed and measured airways pressures not lower than set values. This poses the risk of the user reacting to the alarm message by increasing the set pressure value inappropriately. In recent years there has been growing trends to not intubate preterm infants and use non-invasive respiratory therapies as first line. Morley et al showed that with early non-invasive nasal CPAP compared to invasive ventilation therapy, fewer infants received oxygen at 28 days and they had fewer days of ventilation but with an increase in incidence of pneumothorax (9·1% CPAP, 3·1% Intubated).[15] In this report the type of delivery device was not noted. Makhoul et al reported increased risks of pneumothorax using nCPAP via Aldadin-1 device (Electromedical Equipment, Brighton, England).[16] In preterm animal models as few as 6 excessive inflation pressures initiate severe lung injury.[17] There is potential for lung damage with inadvertent sustained distending pressures and repetitive cycling during neonatal CPAP with partial or total expiratory limb occlusion as a contributing factor.[18] Interaction between ventilator software, mechanics and delivered patient therapy is complex.

The current international safety standards designed to guide manufacturers in minimum safety standards is outdated and does not provide sufficient patient protection particularly in view of newer sophisticated functions and the wide distribution of patient weight in neonatal patients (500gm– 6kg). The ISO standards 10651·1–2004 entitled “Lung ventilators for medical use part 1: Requirements.”[10] simply states “the maximum limited pressure at the patient connection port which may occur during the intended use or under single fault condition shall not exceed 120% of the maximum adjustable pressure”. This guidance does not provide satisfactory limits; nor does it distinguish between invasive intubated ventilator support (via endotracheal tube); or non-invasive naso-pharyngeal pressure support.

## Conclusion

Our results are concerning given that the majority of ventilators tested responded to partial or complete expiratory limb occlusion in potentially hazardous ways. We found that there is a potential to expose the neonatal lung to pressures and volumes that would be clinically unacceptable. Currently, the ISO standards do not guide manufacturers to prevent this potential event. One ventilator tested behaved in an appropriate manner which would suggest it is currently within scope for all manufacturers to implement in future neonatal ventilator design. Of greater concern is the apparent lack of improvement in safety of ventilator inspiratory pressure regulator design over the past thirty years since this problem was first brought to light by Hall et al in 1983.

## Supporting Information

S1 Dataset (XLS)
